# Supplementary figures and images for: TunR2, a novel mode-of-action tunicamycin-type antibiotic: Pharmacokinetics in C57BL/6 mouse and Holstein cattle
Source: PLoS One. 2025 Jul 23;20(7):e0327932. doi: 10.1371/journal.pone.0327932 (PMC12286339; doi:10.1371/journal.pone.0327932)

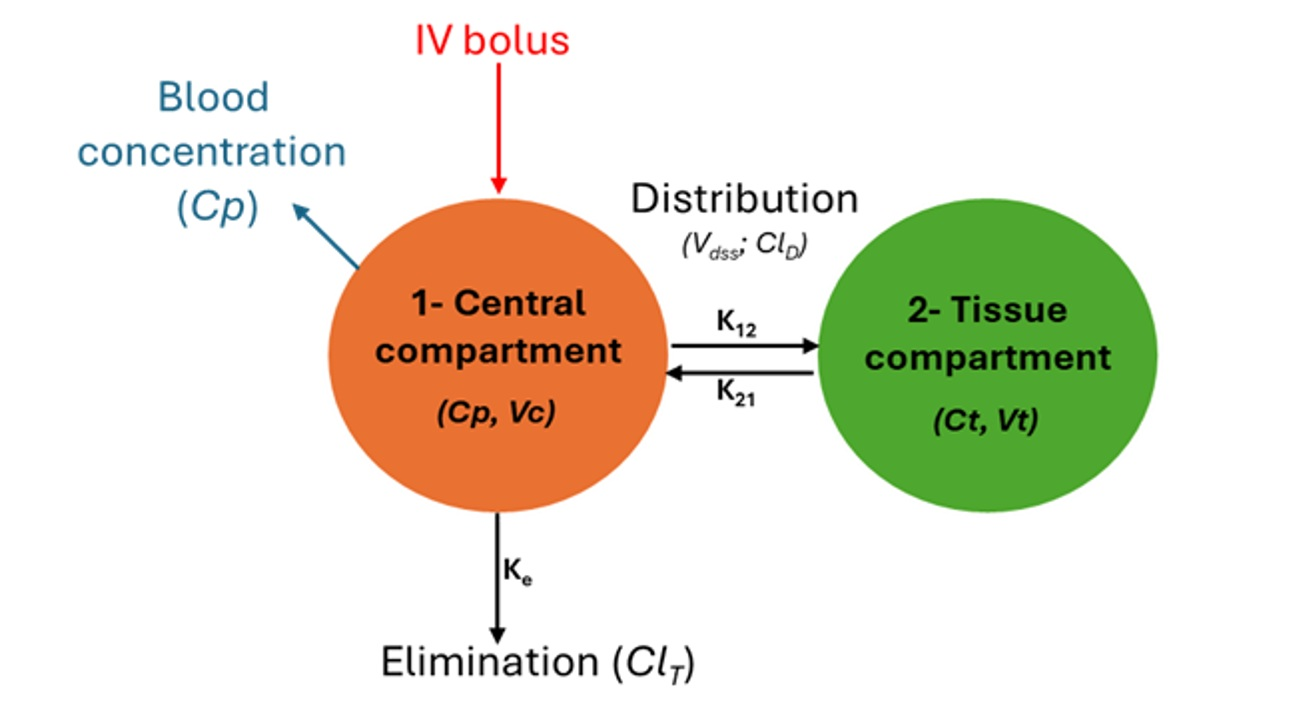

Supplement: S1 Fig — Cp, drug concentration in plasma (central compartment), Ct, drug concentration in tissue compartment; Vc, volume of the central compartment; Vt: volume of the tissue compartment; Vdss, volume of distribution at steady state; K12, rate constant of distribution from central to tissue/peripheral compartment; K21, rate constant of distribution from tissue to central compartment; Ke, elimination rate constant; ClD, rate of distribution/intercompartment; ClT total clearance rate. (TIF) [file pone.0327932.s001.tif]

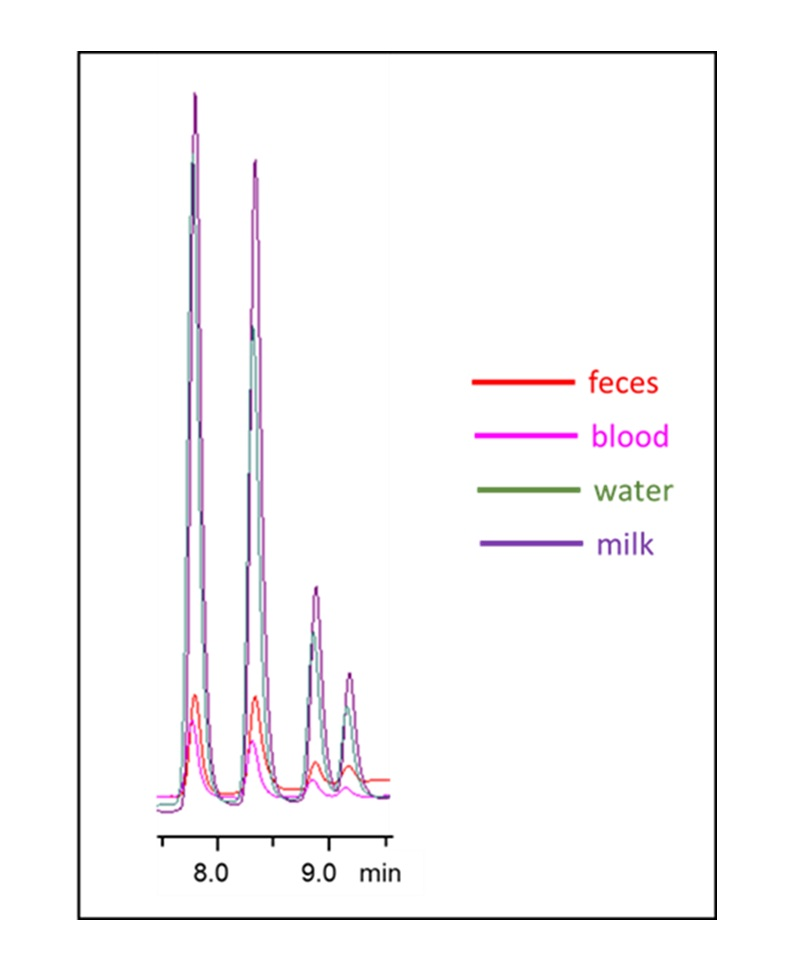

Supplement: S2 Fig — 1ug/mL of TunR2/DOC was added to 1 mL of blood or milk and 1 g of feces to evaluate the sensitivity of detection of the drug, compared to water samples. Dry residues from blood, milk, and water samples were redissolved in 200 μL methanol and feces in 1mL methanol (5x less concentrated compared to the other samples). The TunR2 detection in blood by LC-SIM/MS is reduced in 5-fold (pink line). Drug could not be detected in biological samples with 0.5 ug/mL or less of TunR2. (TIF) [file pone.0327932.s006.tif]

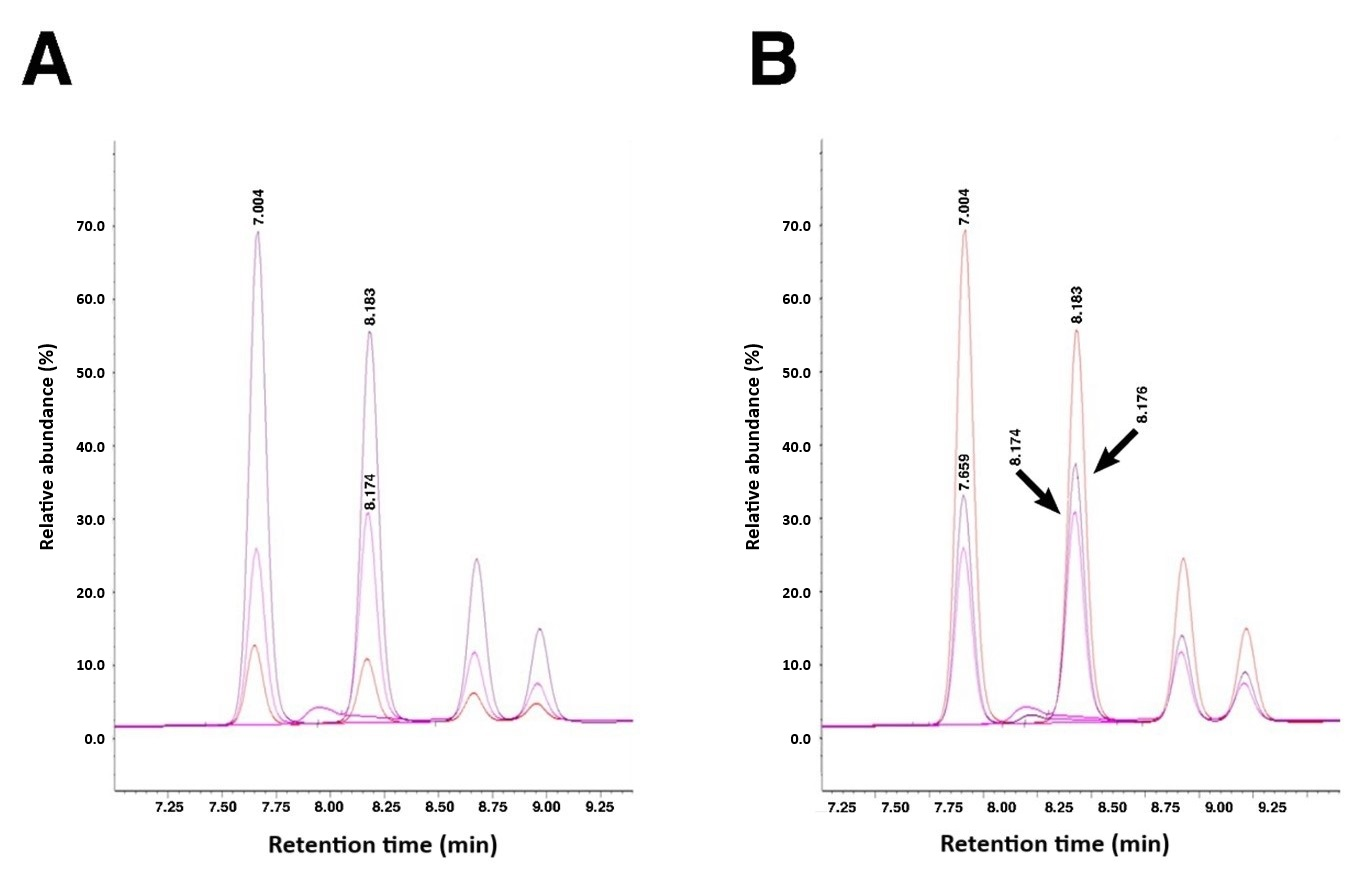

Supplement: S3 Fig — B) TunR2 (1ug/mL) detection in BSA 20 mg/mL (purple line) and 40 mg/mL (pink line) compared to sample in water (red line). Note that LC-ESI-MS has the highest sensitivity for the detection of TunR2 in water, followed by samples containing BSA (20 and 40 mg/mL) and finally FBS. (TIF) [file pone.0327932.s007.tif]

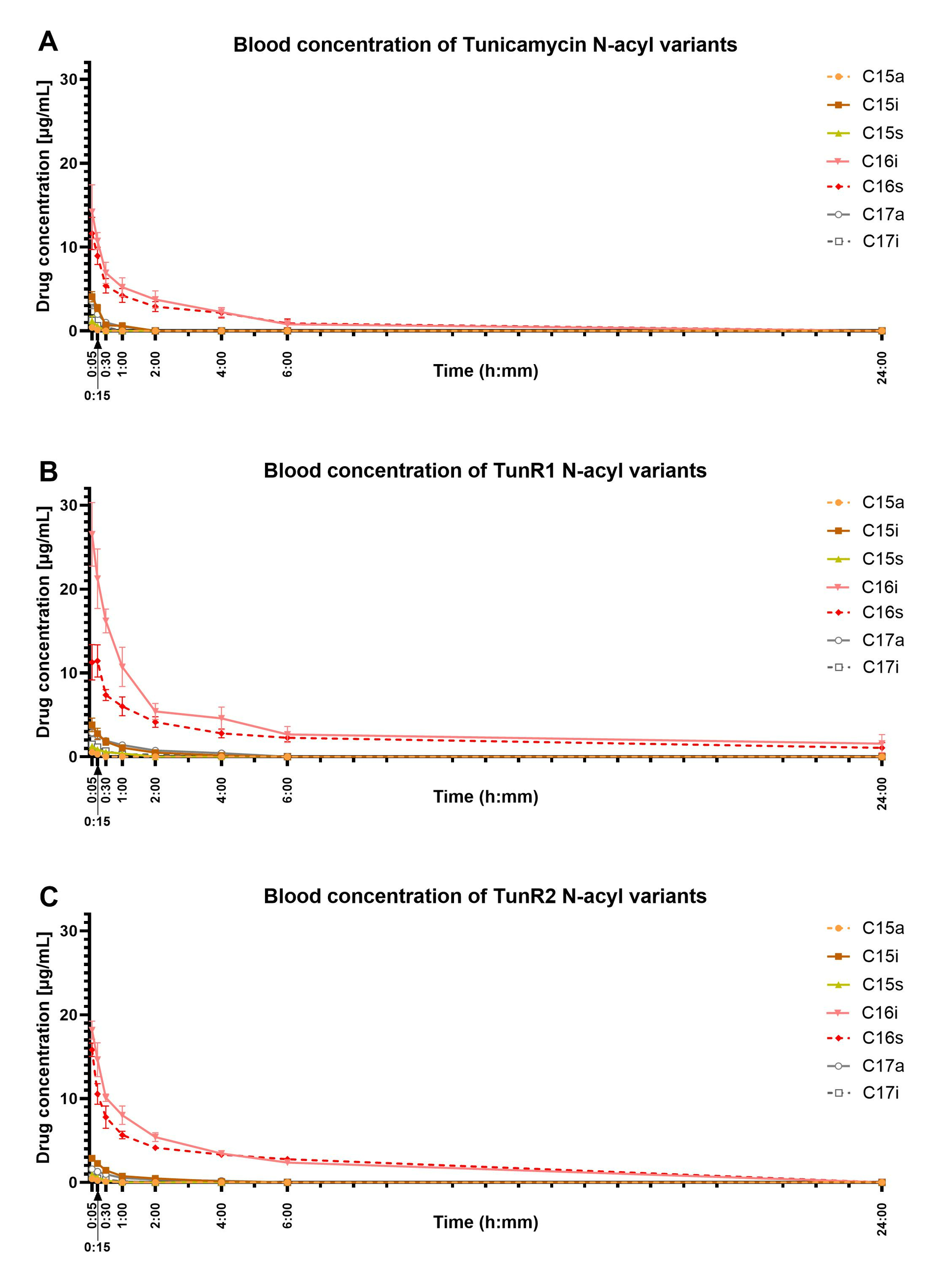

Supplement: S4 Fig — Graphs show the N-acyl variant concentration (µg/mL ± SEM) vs. time. (TIF) [file pone.0327932.s008.tif]

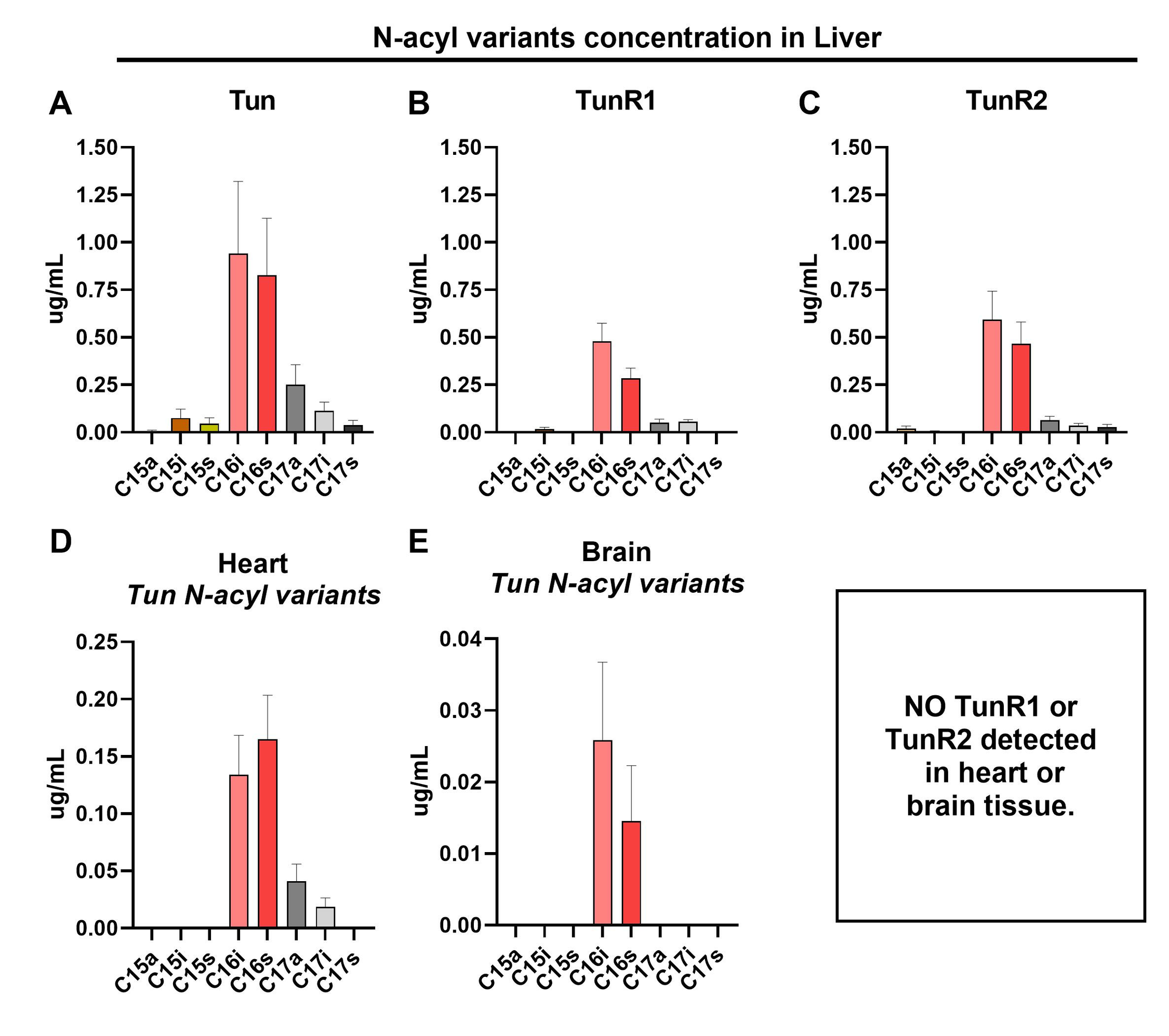

Supplement: S5 Fig — D-E) Comparative concentration of the different N-acyl variants of Tun in the heart and brain. TunR1 and TunR2 were not detected in those tissues. (TIF) [file pone.0327932.s012.tif]

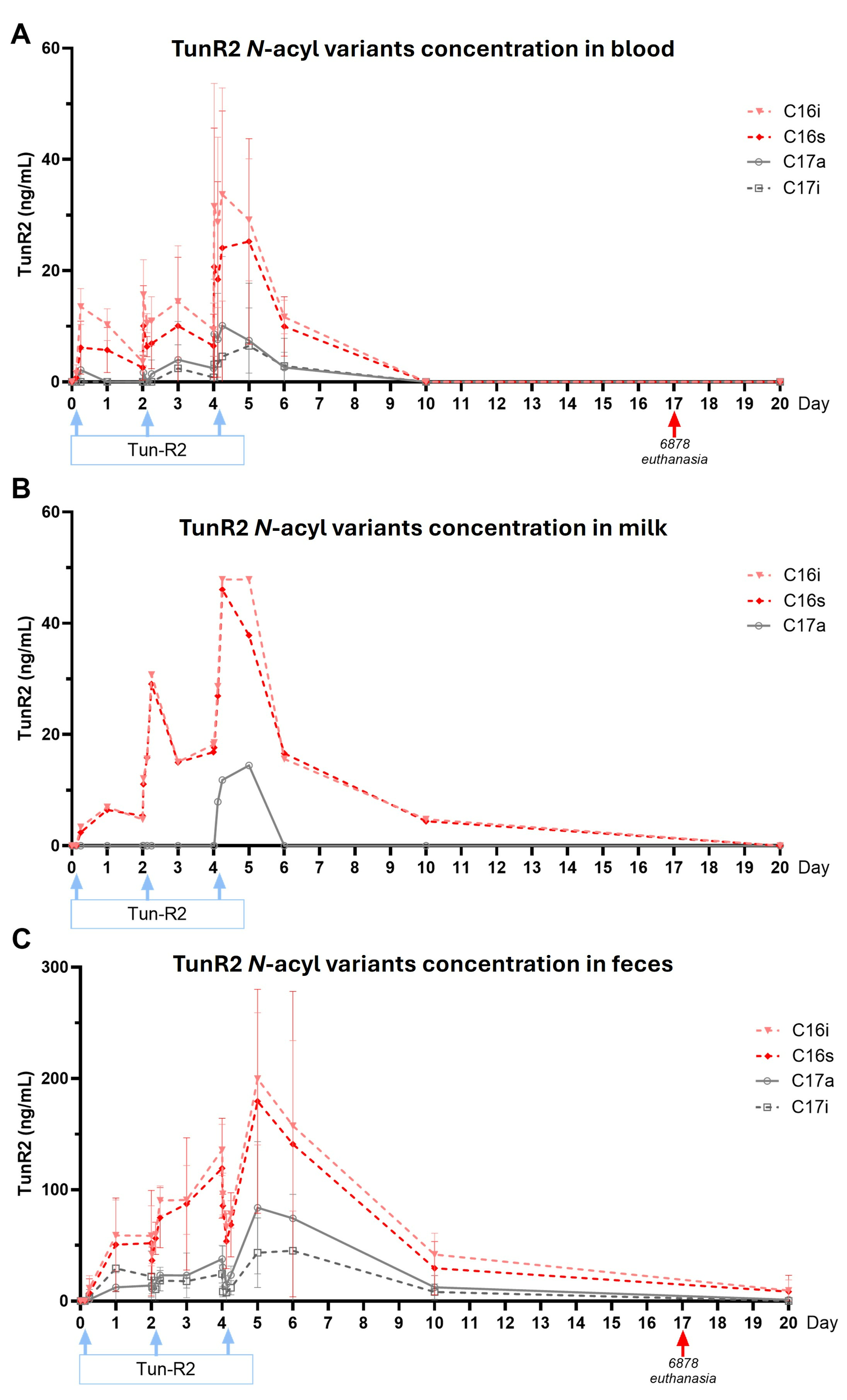

Supplement: S6 Fig — (TIF) [file pone.0327932.s013.tif]

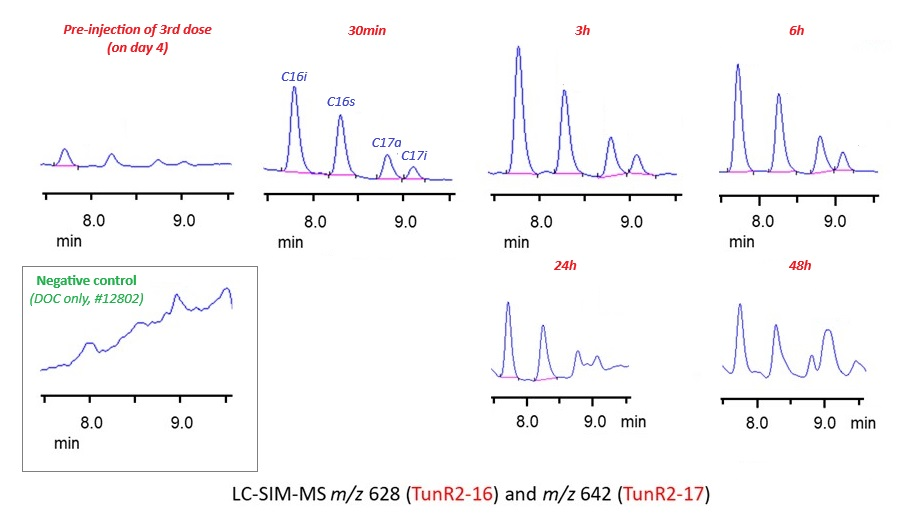

Supplement: S7 Fig — Blood samples from Cow 5327 at different time points:- pre-injection of 3rd dose, 30 min, 3 h, and 6 h on day 4, 24 h (day 5), and 48 h (day 6), showing the chromatographic peaks of the N-acylated TunR2 variants: C16-iso (7.74 min retention time), C16-straight (8.26 min), C17-anteiso (8.78 min), and C17-iso (9.06 min). At the pre-injection of the 3rd dose time point (on Day 4) there is a small residual peak for TunR2-16-iso due to carryover from the prior bolus of the drug. After just 30 min, the four TunR2 components are well defined in the blood stream and reach a maximum at 3 h post-injection. After this time, the different N-acylated forms of the TunR2 drug are cleared at the same rate (that is by 6 h post-injection), and only residual amounts of the two major forms (TunR2-16-iso and TunR2-16-straight) are still biologically available in the bloodstream after 24–48 h. Similar kinetics were also observed for Cows 6739 and 6878, and no HPLC Tun components were apparent for the negative control animal (cow 12802 which received only DOC) as it shows in the graph inside the gray box shows the negative control (cow 12802) at 24 h on Day 5. LC peaks were monitored by SIM-MS of TunR2-16 (m/z 628) and TunR2-17 (m/z 642). (TIF) [file pone.0327932.s014.tif]

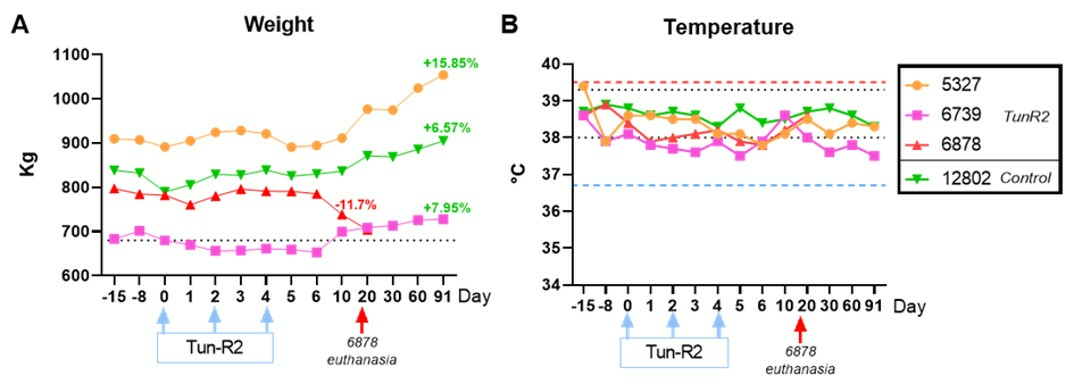

Supplement: S8 Fig — (A) The weights of each animal in kilograms (kg) over the 91-day study. Note that the normal weight of an adult Holstein cow is approximately 680 kg (dotted line). The graph also displays the percent change in weight for each animal, with increases shown in green and decreases in red. (B) Rectal temperature (°C). Normal rectal temperature for a dairy cow is 38–39.3°C (black dotted lines). Values >39.5°C or <36.7°C are considered hyper and hypothermia (dashed lines red and blue). (TIF) [file pone.0327932.s015.tif]

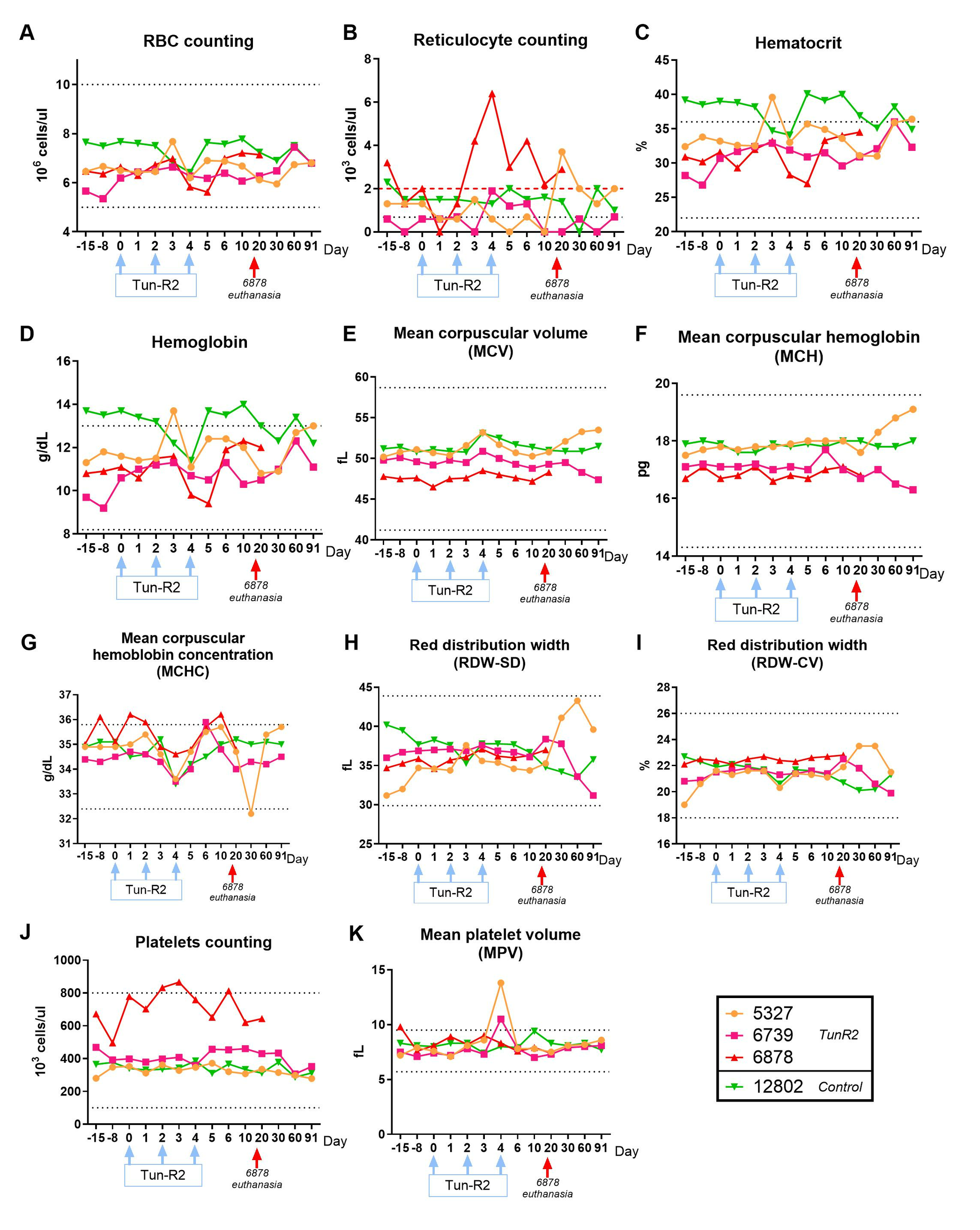

Supplement: S9 Fig — Erythrogram and Thrombogram. (A) Red blood cell (RBC) counts. (B) Reticulocyte counting (immature RBC). Dashed red line marks the limit of values that are considered significantly high and indicates increased erythropoiesis in animal 6878. This animal is not suffering from anemia, so the cause of a high reticulocyte count may be due to systemic disease (cardiac, pulmonary) or even an increase in erythropoietin production due to localized hypoxia in the kidney, which may or may not be due to subclinical renal disease. (C) Hematocrit. An elevated hematocrit with a normal RBC count in the control animal indicates possible dehydration because it is accompanied by a slight increase in total plasma protein (see Figure 9). (D) Hemoglobin, with a pattern similar to the hematocrit. (E) Mean corpuscular or cell volume (MCV). This value represents the volume of the average RBC, that in this case all animals are normocytic. Mean corpuscular or cell hemoglobin (MCH) indicate the average amount of hemoglobin in the RBC, that in this case all animals are normochromic. (F) Mean corpuscular hemoglobin concentration (MCHC), this value depends on the hemoglobin concentration and hematocrit. (H) Red distribution width (RDW)-standard deviation (SD) and (I) RDW- coefficient of variation (CV) reflect the cell volume variation within the RBC, higher values indicate more variation. (J) Platelets counting. Cow 6878 has the highest values, close to normal limit. (K) Mean platelet volume (MPV) reflects the average volume or size of platelets but also could be increased by platelet clumps. At the bottom right are the legends for all figures. Normal values are between black dotted lines. For all panels, the TunR2 doses are indicated with light blue arrows and cow 6878 euthanasia prior to study endpoint is indicated with a red arrow. Absolute eosinophils and basophils count were normal in all animals (TIF) [file pone.0327932.s016.tif]

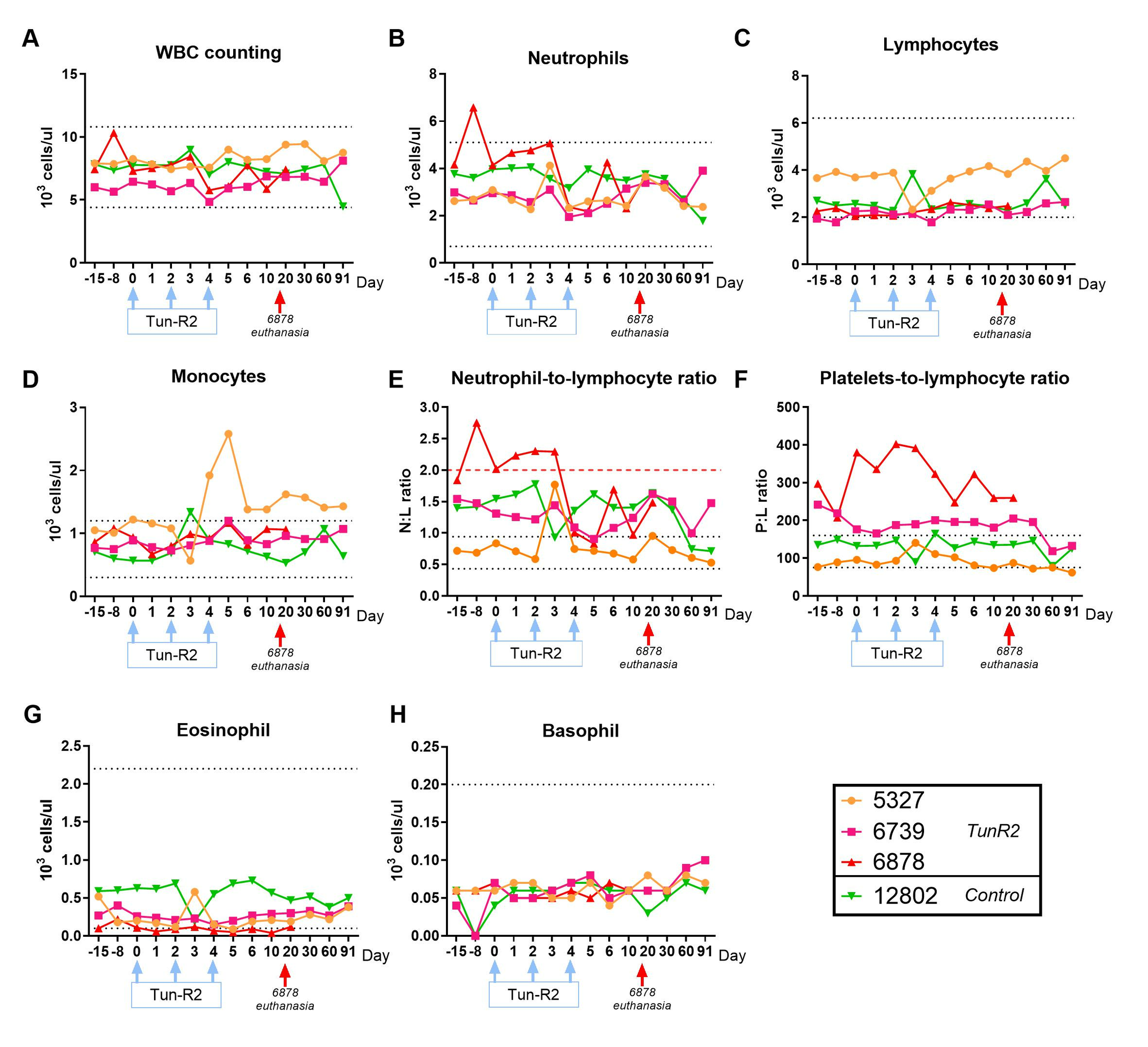

Supplement: S10 Fig — (A) White blood cells (WBC) counting. (B) Absolute neutrophil count. (C) Absolute lymphocyte count. (D) Absolute monocyte count. Monocytosis without neutrophilia in animal 5327 could be caused by a healing process of a chronic infection. (E) Neutrophil to lymphocyte ratio (N:L ratio). Red dashed line shows the limit of a significant increase in neutrophils (ratio >2). (F) Platelets to lymphocyte ratio (P:L ratio). Both the elevated N:L and P:L ratio indicate a possible inflammatory response in animal 6878 [20]. (G) Absolute eosinophils count and (H) absolute basophils count were normal in all animals. At the bottom right is the legends for all figures. Normal values are between black dotted lines. For all panels, the TunR2 doses are indicated with light blue arrows and cow 6878 euthanasia prior to study endpoint is indicated with a red arrow. Absolute eosinophils and basophils count were normal in all animals. (TIF) [file pone.0327932.s017.tif]

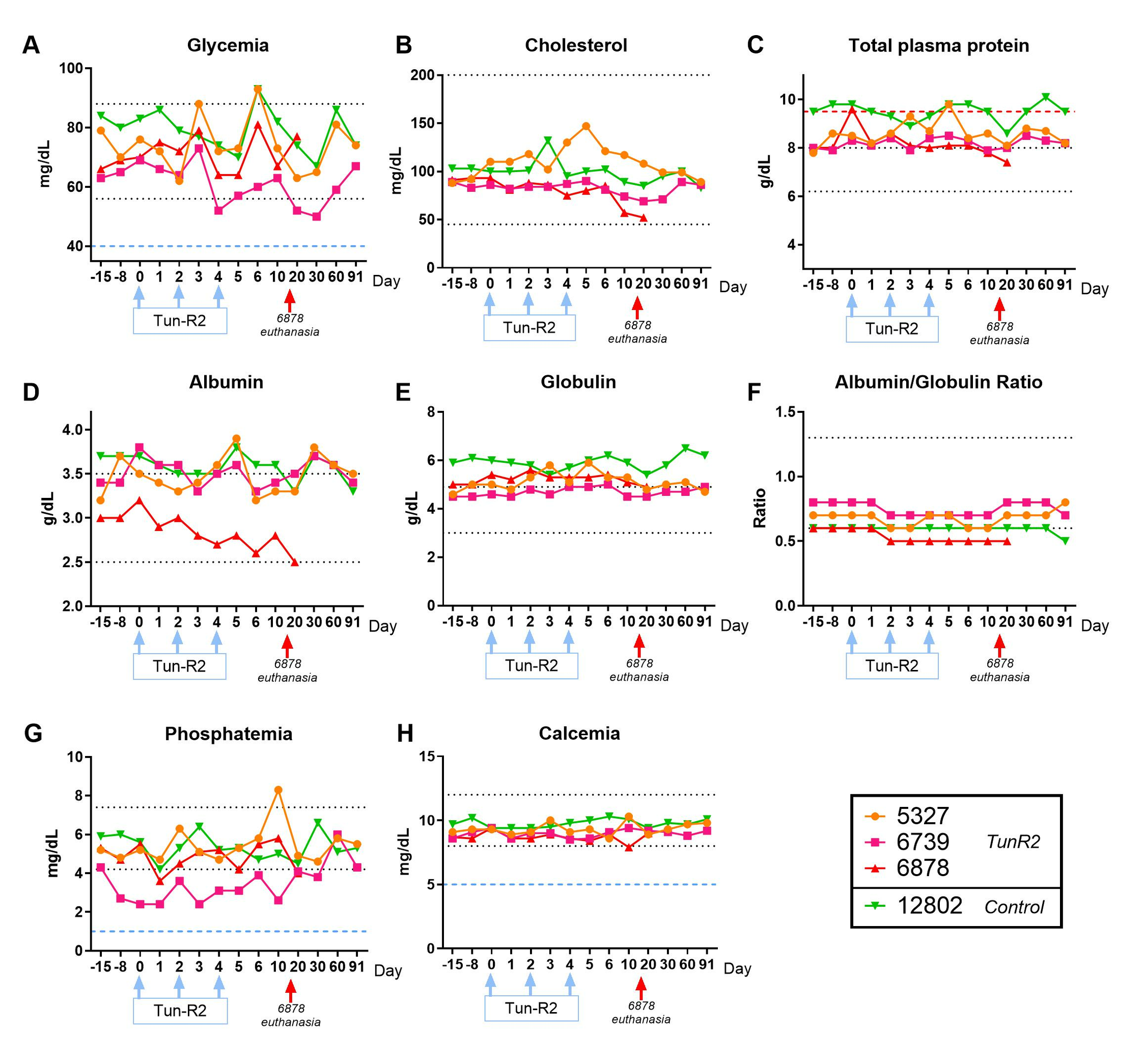

Supplement: S11 Fig — Related to energy metabolism: (A) Blood glucose concentration (glycemia) and (B) Cholesterol. Not significant decrease in glucose and cholesterol detected. Proteins in blood: (C) Total plasma protein, (D) Albumin, (E) Globulin, and (F) Albumin-globulin ratio. Panhyperproteinemia could be dehydration and a slightly increase in globulins due to an inflammatory response (hyperglobulinemia). Minerals: (G) Phosphate in blood (phosphatemia) and (H) Calcium in blood (calcemia). Lower levels of albumin (in cow 6878) or phosphate (cow 6739) could indicate loss by enteropathy. At the bottom right is the legends for all figures. Normal values are between black dotted lines. Red and blue dashed line shows the limit of a significant increase or decrease in the value of the analyte in blood. For all panels, the TunR2 doses are indicated with light blue arrows and cow 6878 euthanasia prior to study endpoint is indicated with a red arrow. (TIF) [file pone.0327932.s018.tif]

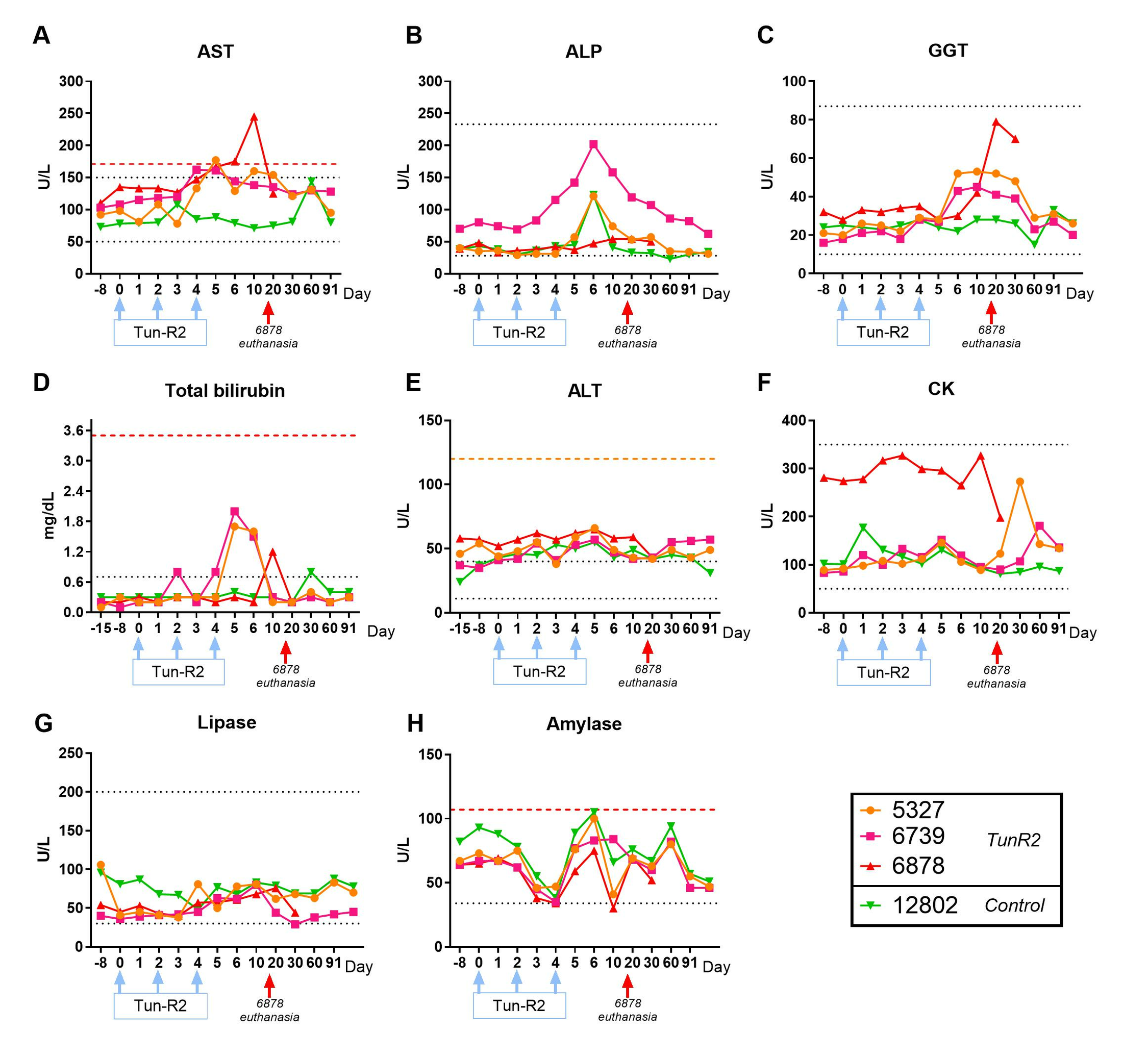

Supplement: S12 Fig — Related to liver function: (A) Aspartate aminotransferase (AST). No significant increase detected (need to be above the red dashed line), except for cow 6878 at time pre-euthanasia (10 days). This enzyme could be increased due to muscle injury if it’s accompanied with an increase in CK. (B) Alkaline phosphatase (ALP). Slight transitional increase at 24–48hs post last dose but remains within normal values. (C) Gamma-glutamyltransferase (GGT). (D) Total bilirubin. Slight transitional increase at 24–48hs post last dose. Other: (E) Alanine aminotransferase (ALT). Not significant increase detected (need to be above the orange dashed line). NOTE: ALT is not a useful indicator of liver disease in large animals due to low enzyme activity in liver tissue. Related to cardiac and skeletal muscle: (F) Creatine Kinase (CK). Also need to consider AST levels. Related to exocrine pancreatic function: (G) Lipase and (H) Amylase.At the bottom right is the legends for all figures. Normal values are between black dotted lines. Red dashed line shows the limit of a significant increase in the value of the analyte in blood. For all panels, the TunR2 doses are indicated with light blue arrows and cow 6878 euthanasia prior to study endpoint is indicated with a red arrow. (TIF) [file pone.0327932.s019.tif]

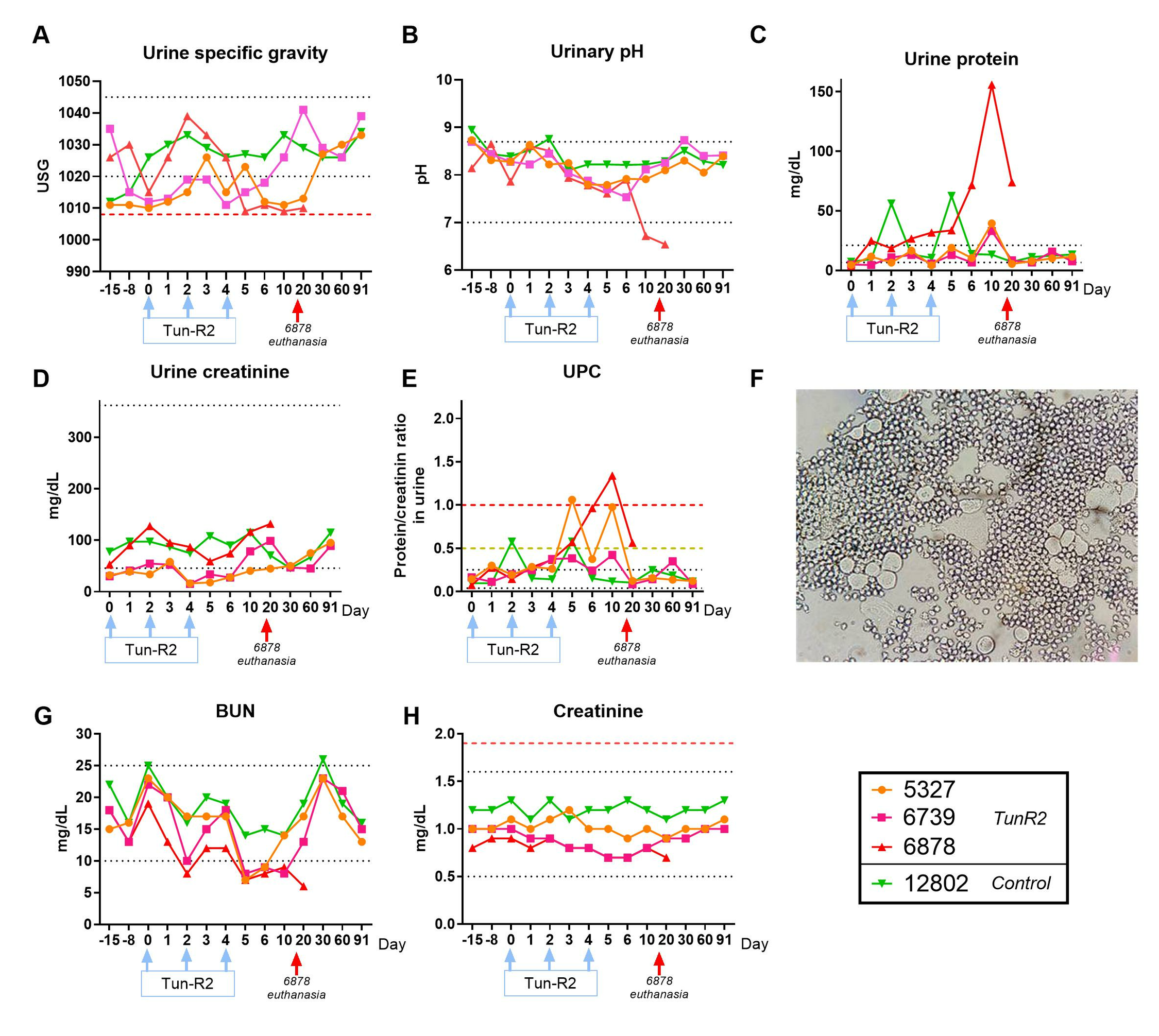

Supplement: S13 Fig — (A) Urine specific gravity (USG). Values between bottom dotted line and red dashed line is isosthenuria (not urine concentration or dilution), may indicate kidney damage if accompanied by proteinuria (see graph E-UPC) and the presence of tubular cells or cast in the urinary sediment. (B) Urinary pH. Only a significant decrease detected in cow 6878 at time pre-euthanasia (10 days). (C) Urine protein needs to be evaluated with (D) Urine creatinine using the (E) Urine protein to creatinine ratio (UPC). Slight transitional increase at 1–5 days post last dose, that could indicate mild and reversible damage, since the values then normalize, together with the USG. (F) Representative photo of Urinary sediment (40x) cow #12802 at day 5. The abundant presence of leukocytes (>100 cells per high power field/microscopy field at 400x (hpf)) pyocites and transitional epithelial cells can be observed. This animal had a urinary infection between day 1–10. Also, cow 6878 presented a milder urinary tract infection between day 0–3 and after day 10 (See Table S1). (G) Blood urea nitrogen (BUN) and (H) Creatinine showed normal levels. The slight transitional decrease in BUN could be due to an increase in the urea excretion by urine.At the bottom right is the legends for all figures. Normal values are between black dotted lines. Red dashed line shows the limit of a significant increase in the value of the analyte in blood. For all panels, the TunR2 doses are indicated with light blue arrows and cow 6878 euthanasia prior to study endpoint is indicated with a red arrow. (TIF) [file pone.0327932.s020.tif]

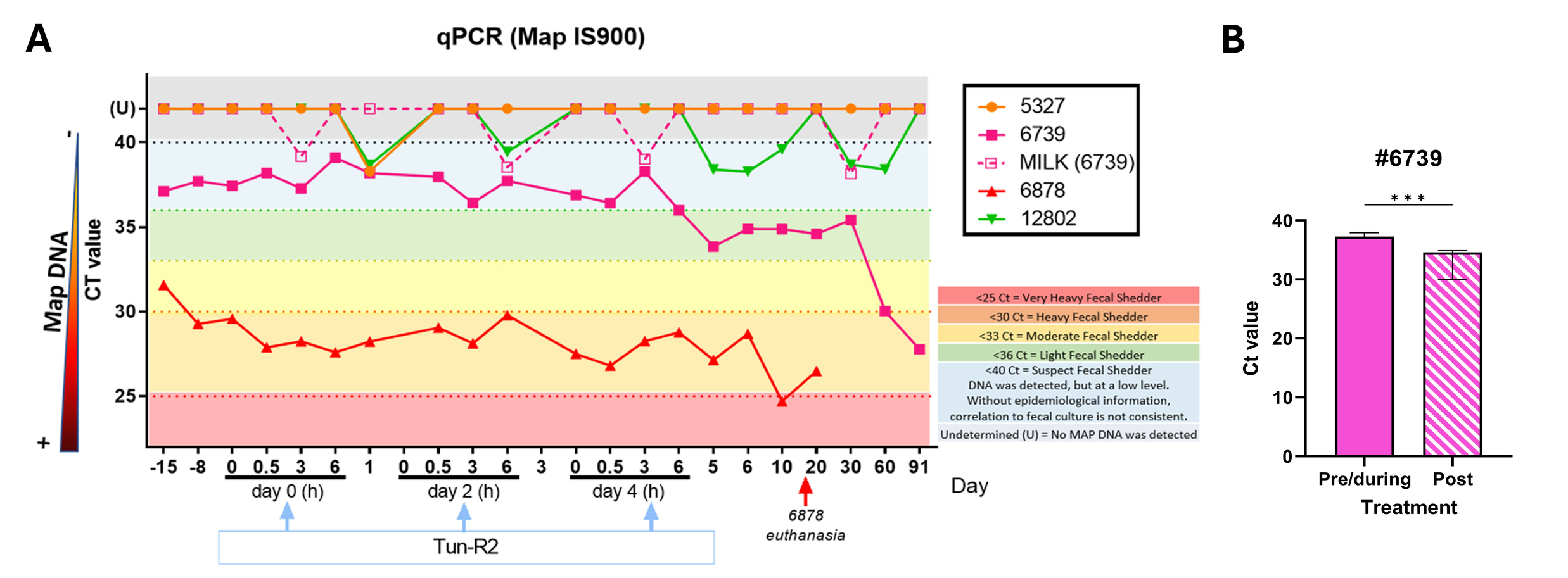

Supplement: S14 Fig — Note the reddish tissue of #6878 indicative of inflammation. (TIF) [file pone.0327932.s021.tif]

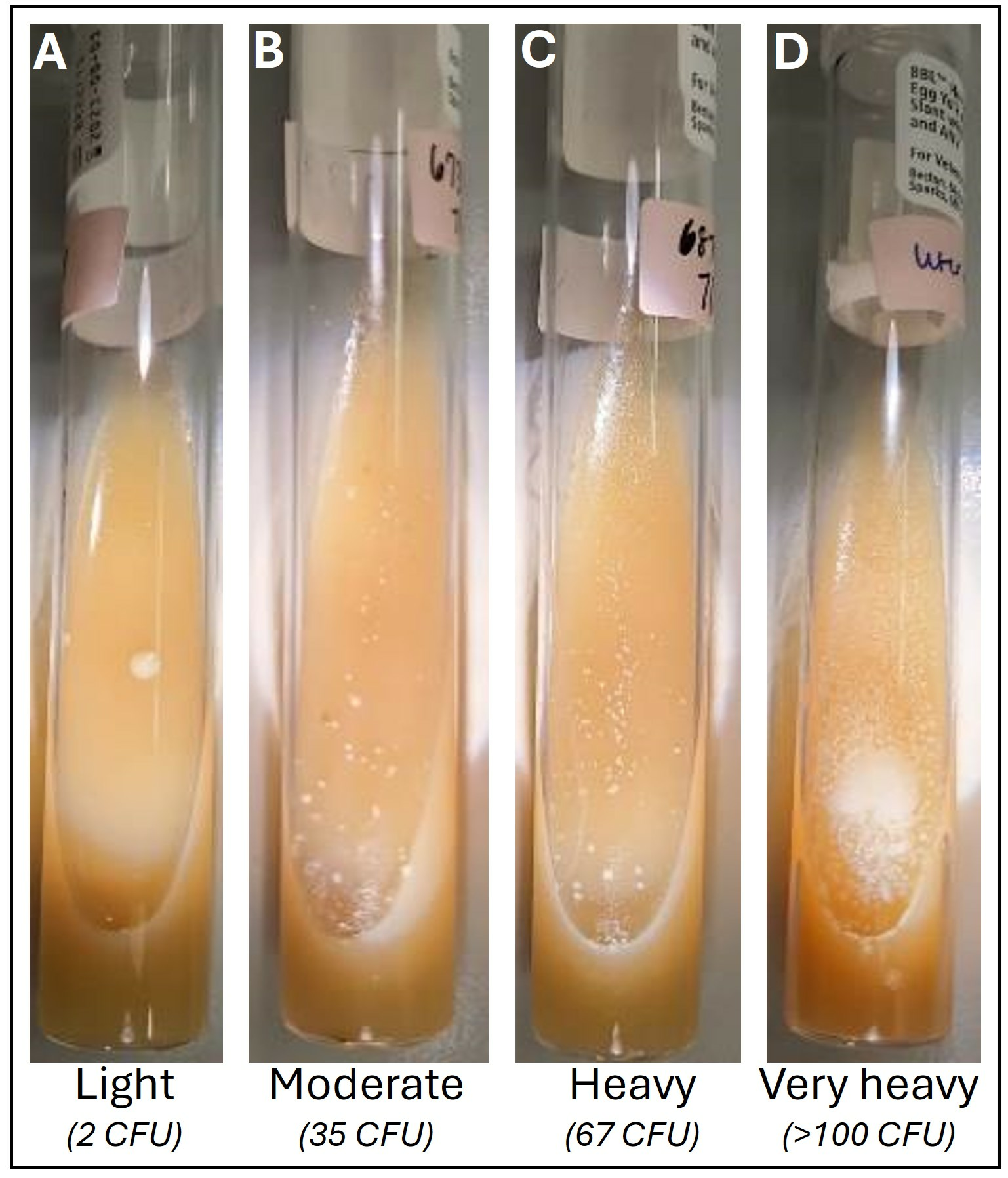

Supplement: S15 Fig — (A) Ileum (H&E, 10x) from TunR2 treated cow #6878 with diffuse intermediate-lymphocytic lesion with crypt abscesses. Bar = 200 µm (B) Jejunum (ZN, 60x) from TunR2 treated cow #6878 showing the presence of acid-fast bacteria in epithelioid macrophages. Bar = 50 µm. (C) Proximal ileum (H&E, 20x) from TunR2 treated cow #6739 with diffuse multibacillary lesion. Bar = 100 µm. (D) Jejunum (H&E, 40x) from control (DOC treated) #12802 with eosinophilic enteritis and less severe lesions of Johne’s Disease focal. Bar = 50 µm. (TIF) [file pone.0327932.s022.tif]

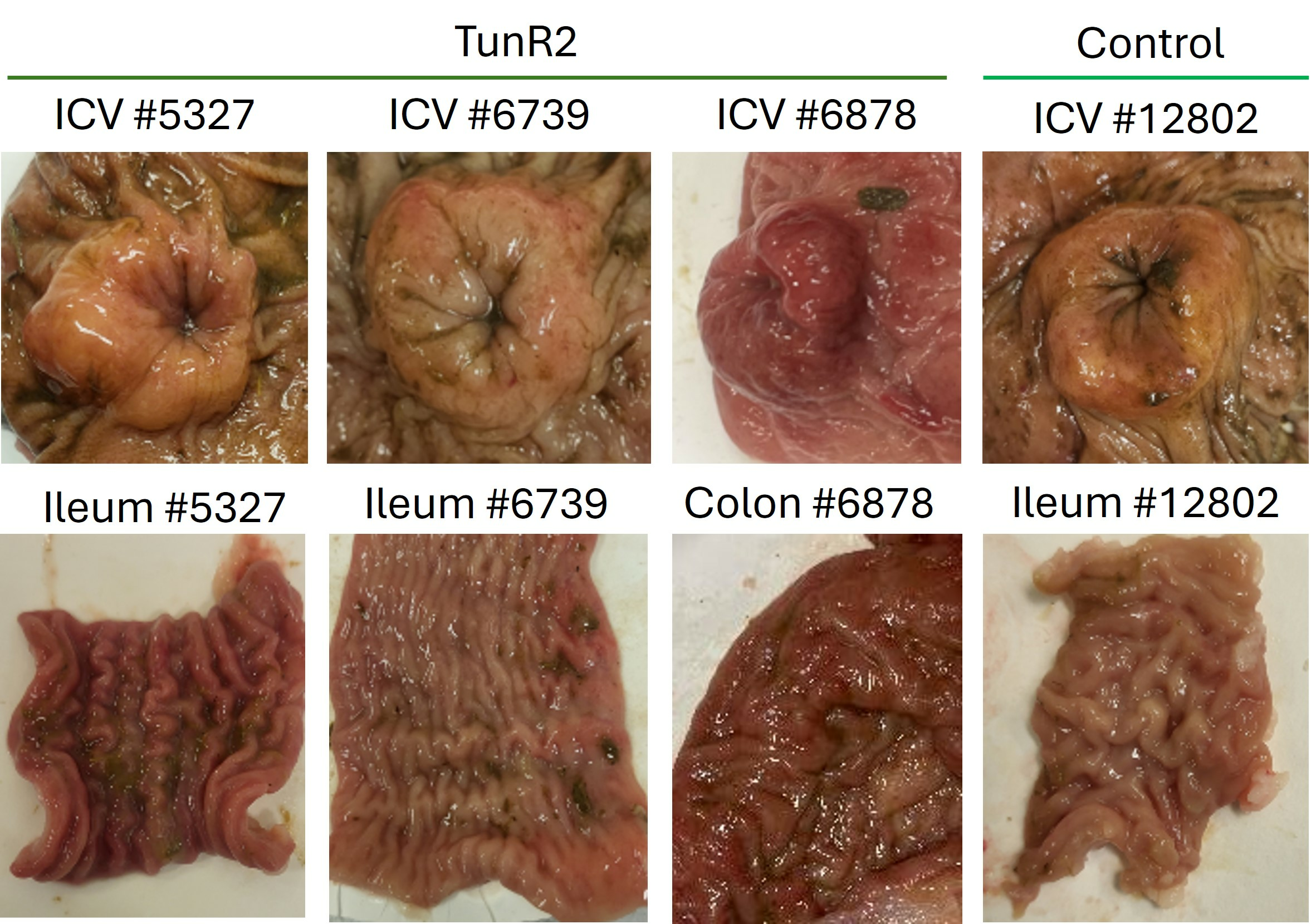

Supplement: S16 Fig — (A) Control cow without Johne’s disease showing normal liver tissue (20x). Bar = 100 µm. (B) Cow 5466 with JD (no treatment with TunR2 or DOC) showing vacuolar degeneration of hepatocytes (20x). Bar = 100 µm. (C) TunR2 treated cow #6878 showing vacuolar degeneration of hepatocytes (40x). Bar = 50 µm. (D) Control (DOC treated) showing swollen enlarged hepatocytes (20x). Bar = 100 µm. (E) Morphology of portal tracts in TunR2 treated cow 6878 that shows moderate periportal fibrosis (20x). Bar = 100 µm. (F) TunR2 treated cow #6739 showing a lymphoplasmacytic pericholangitis/portal hepatitis (20x). Bar = 100 µm. (TIF) [file pone.0327932.s023.tif]

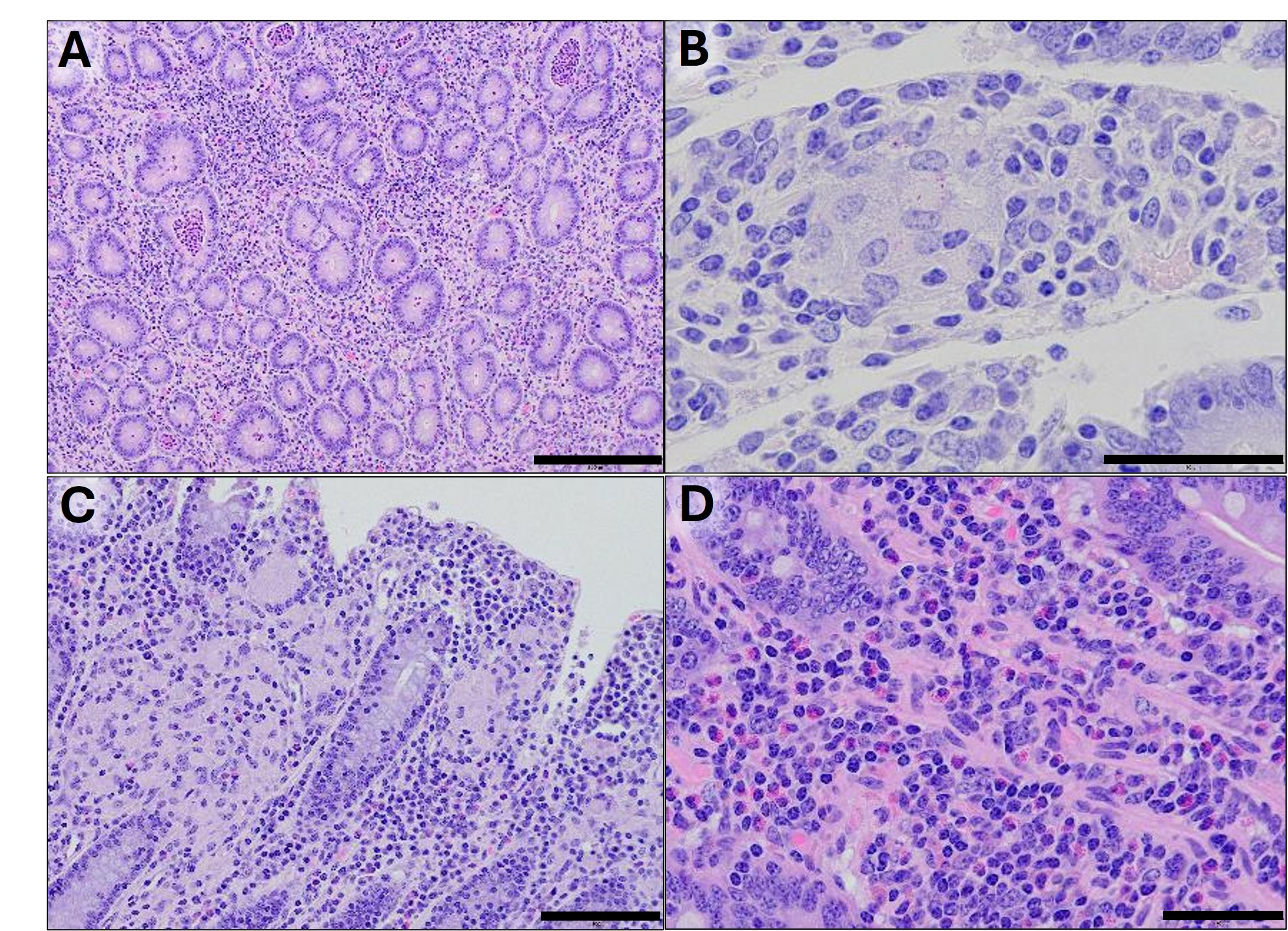

Supplement: S17 Fig — (A) TunR2 treated cow #5327 showing mild membranous glomerulonephritis with attenuated tubular epithelium (10x). Bar = 200 µm. Upper right corner: lymphoplasmacytic interstitial nephritis (20x). Bar = 100 µm. (B) TunR2 treated cow #6739 showing mild membranous glomerulonephritis (40x). Bar = 50 µm. (C) TunR2 treated cow #6878 showing mild lymphoplasmacytic interstitial nephritis (10x). Bar = 200 µm. (D) Control (DOC treated) cow #12802 showing moderate lymphoplasmacytic interstitial nephritis (10x). Bar = 200 µm. Upper right corner: mild neutrophilic tubular nephritis (20x). Bar = 100 µm. (TIF) [file pone.0327932.s024.tif]

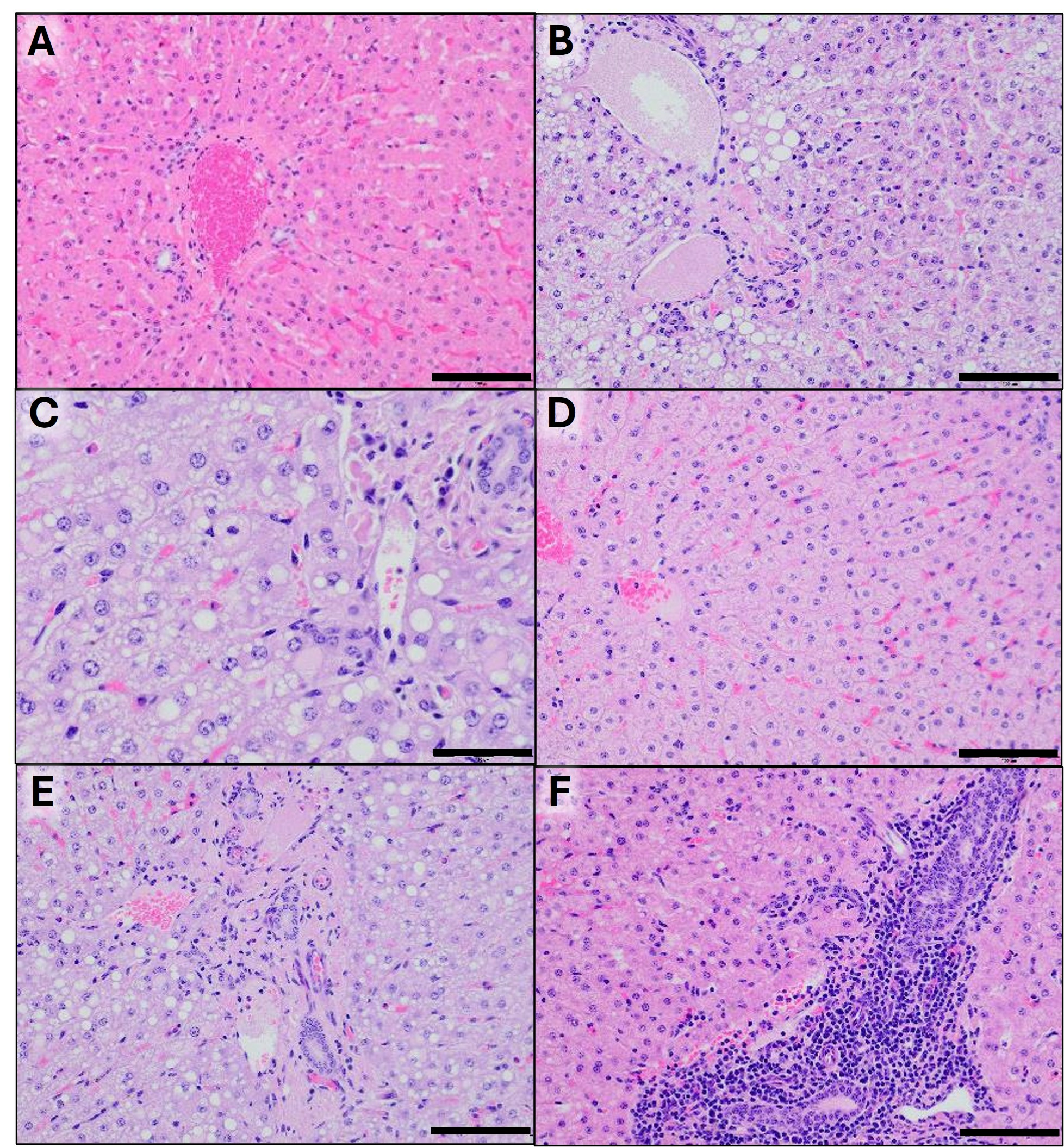

Supplement: S18 Fig — The graph shows the Ct values for IS900 qPCR (y-axis) on the different time point (in days, x-axis). The TunR2 doses are indicated with light blue arrows and cow 6878 euthanasia prior to study endpoint is indicated with a red arrow (on day 18). The graph shading key is shown at the lower right. Note that both milk (dashed line) and feces were tested for cow 6739. B- IS900 qPCR fecal Ct values for cow #6739. Data expressed as Ct (median ± interquartile range) from pre/during the treatment (days −15 to 5) and post treatment (days 6 to end point). Statistical analysis was performed using the non-parametric test Mann-Whitney (***p < 0.001). No significant differences were observed in the rest of the animals. (TIF) [file pone.0327932.s026.tif]

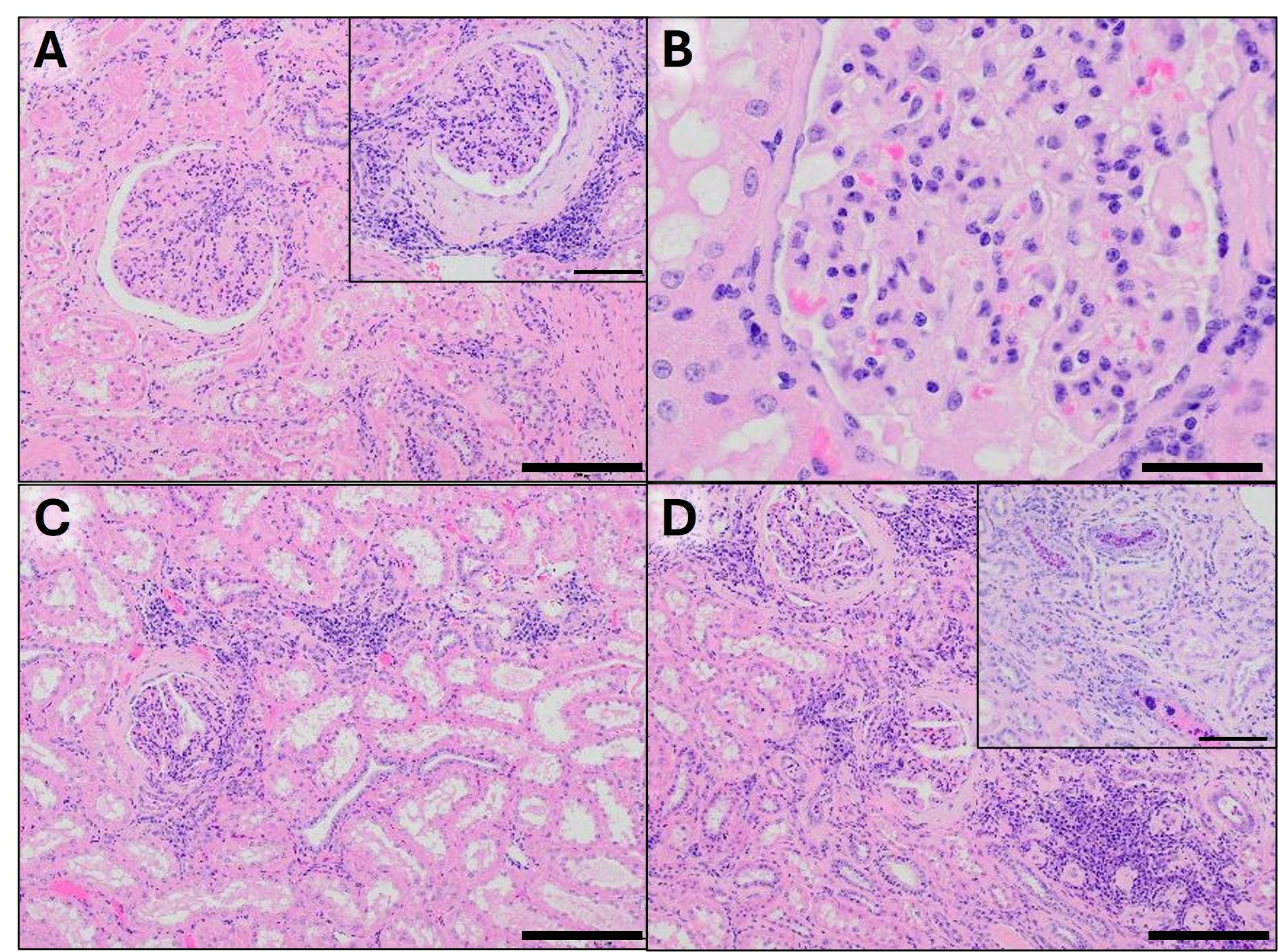

Supplement: S19 Fig — A-Cow 6739-ovaries, B-Cow 6739 feces at day 60, C-Cow 6878 feces at day 0, and D-6878 uterus. (TIF) [file pone.0327932.s028.tif]
