# Supplementary material for: TunR2, a novel mode-of-action tunicamycin-type antibiotic: Pharmacokinetics in C57BL/6 mouse and Holstein cattle
Source: PLoS One. 2025 Jul 23;20(7):e0327932. doi: 10.1371/journal.pone.0327932 (PMC12286339; doi:10.1371/journal.pone.0327932)
Supplement: S1 Table — (DOCX) [file pone.0327932.s002.docx]

**S1 Table. First mouse trial**

| **Group** | **N** | | **Treatment** | **Dose (mg/mL)** |
| --- | --- | --- | --- | --- |
| 1 | 8(4M/4F) | | DMSO | N/A |
| 2 | 8(4M/4F) | | Tunicamycin | 0.2 |
| 3 | 8(4M/4F) | | Tunicamycin | 2 |
| 4 | 8(4M/4F) | | Tunicamycin | 10 |
| 5 | 8(4M/4F) | | TunR1 | 0.2 |
| 6 | 8(4M/4F) | | TunR1 | 2 |
| 7 | 8(4M/4F) | | TunR1 | 10 |
| 8 | 8(4M/4F) | | TunR2 | 0.2 |
| 9 | 8(4M/4F) | | TunR2 | 2 |
| 10 | 8(4M/4F) | | TunR2 | 10 |
| Each animal received a single 30uL intravenous bolus dose | | | | |
| M: male. F: female | |  | |  |
