# Supplementary material for: TunR2, a novel mode-of-action tunicamycin-type antibiotic: Pharmacokinetics in C57BL/6 mouse and Holstein cattle
Source: PLoS One. 2025 Jul 23;20(7):e0327932. doi: 10.1371/journal.pone.0327932 (PMC12286339; doi:10.1371/journal.pone.0327932)
Supplement: S2 Table — (DOCX) [file pone.0327932.s003.docx]

**S2 Table.** **Ratio of the different compounds (carbon chain length) and *N*-acyl variants (*anteiso*, *iso*, and *straight*) in Tun, TunR1 and TunR2, expressed in percentage (%)**

| **Compound** | | **Tun** | **TunR1** | **TunR2** |
| --- | --- | --- | --- | --- |
| C14 | | 3.7 | 2.8 | 3.7 |
| C15 | *Anteiso* | 4.1 | 2.9 | 4.1 |
|  | *Iso* | 12.8 | 10 | 12.8 |
|  | *Straight* | 4.3 | 3.7 | 4.3 |
|  | TOTAL | 21.2 | 16.6 | 21.2 |
| C16 | *Iso* | 30.7 | 42.3 | 30.7 |
|  | *Straight* | 24.8 | 21 | 24.8 |
|  | TOTAL | 55.5 | 63.3 | 55.5 |
| C17 | *Anteiso* | 12.5 | 10.5 | 12.5 |
|  | *Iso* | 7.1 | 6.8 | 7.1 |
|  | TOTAL | 19.6 | 17.3 | 19.6 |
