# Supplementary material for: TunR2, a novel mode-of-action tunicamycin-type antibiotic: Pharmacokinetics in C57BL/6 mouse and Holstein cattle
Source: PLoS One. 2025 Jul 23;20(7):e0327932. doi: 10.1371/journal.pone.0327932 (PMC12286339; doi:10.1371/journal.pone.0327932)
Supplement: S3 Table — (DOCX) [file pone.0327932.s004.docx]

**S3 Table.** **Second mouse trial: co-administration of oxacillin with Tun, TunR1 or TunR2**

| **Group** | **N** | **Treatment** | **Dose (mg/mL)** |
| --- | --- | --- | --- |
| 1 | 10(5M/5F) | Oxacilin | 6.7 |
| 2 | 10(5M/5F) | Tun + Oxacilin | 6.7 + 6.7 |
| 3 | 10(5M/5F) | TunR1 + Oxacilin | 6.7 + 6.7 |
| 4 | 10(5M/5F) | TunR2 + Oxacilin | 6.7 + 6.7 |
| Each animal received a single 30uL intravenous bolus dose  M: male. F: female | | | |
