# Supplementary material for: TunR2, a novel mode-of-action tunicamycin-type antibiotic: Pharmacokinetics in C57BL/6 mouse and Holstein cattle
Source: PLoS One. 2025 Jul 23;20(7):e0327932. doi: 10.1371/journal.pone.0327932 (PMC12286339; doi:10.1371/journal.pone.0327932)
Supplement: S4 Table — (DOCX) [file pone.0327932.s005.docx]

**S4 Table.** **General characteristics of cows enrolled in this study**

| **Cow number** | **Treatment^a^** | **Age^b^** | **JD indicators at pre-treatment time** | | | **Weight (kg)** | **BSA^f^ (m2)** | **TunR2 dose (ug/kg)** | | |
| --- | --- | --- | --- | --- | --- | --- | --- | --- | --- | --- |
|  |  |  | **Antibody^c^** | **IFN-g^d^** | **Fecal PCR (Ct)^e^** |  |  | **1^st^** | **2^nd^** | **3^rd^** |
| 5327 | TunR2 | 8y 7m | + | + | U | 908.5 | 6.8 | 110.3 | 55.1 | 49.6 |
| 6739* | TunR2 | 6y 3m | + | + | 29.1 | 692.2 | 5.8 | 142.7 | 71.3 | 64.2 |
| 6878^#^ | TunR2 | 6y 0m | + | + | 29.3 | 791 | 6.3 | 127.4 | 63.7 | 57.3 |
| 12802 | DOC only | 10y 5m | + | + | U | 835.5 | 6.3 | NOT treated | | |
| 5466 | None | 8y 4m | + | + | 37.3 | ND | ND |  |  |  |
| 2222 | None | 7y 2m | + | + | 22.8 | ND | ND |  |  |  |
| 1307 | None | 4y 1m | - | - | 39.3 | ND | ND |  |  |  |
| 1422c | None | 7y 1m | - | - | U | ND | ND |  |  |  |

***** Cow 6739 was the only one lactating.

**^#^** Cow 6878 presented signs of JD and due to the advanced stage of the disease had to be culled on day 17 of the trial.

**^a^**Treatment-Animals received 3 injections of TunR2 in deoxycholate (DOC), or DOC only (#12802) or no injections (histopathology controls).

**^b^**Age-shown in years (y) and months (m) at the time the study began.

**^c^**Antibody-tested for Map antibodies using the IDEXX ELISA test with sample-to-positive ratios greater than 0.60 considered positive.

**^d^**IFN-gamma-used the bovigam test with OD greater than 0.1 considered positive.

**^e^**Fecal PCR-IS900 PCR test with cycle threshold values (Ct) less than 40 considered suspect and less than 36 considered positive.

**^f^**BSA = Body surface area was calculated using the equation 0.14 x W0.57, where W = weight in kilograms (Berman 2003).

U: undetermined (Ct > 40). ND: not determined.
