# Supplementary material for: TunR2, a novel mode-of-action tunicamycin-type antibiotic: Pharmacokinetics in C57BL/6 mouse and Holstein cattle
Source: PLoS One. 2025 Jul 23;20(7):e0327932. doi: 10.1371/journal.pone.0327932 (PMC12286339; doi:10.1371/journal.pone.0327932)
Supplement: S5 Table — (DOCX) [file pone.0327932.s009.docx]

**S5 Table. Comparative pharmacokinetics of Tun homologs in mouse blood.**

| **PK parameters** | **Tun-C15** | **Tun-C16** | **Tun-C17** |
| --- | --- | --- | --- |
| Cp_0 =_ C_max_  (μg/mL ± SD) | 6.7 ± 2.4  **N:** 2.31 ± 0.5 | 27.4 ± 2.8  **N:** 3.6 ± 0.2**^a, b^** | 6.7 ± 2.6  **N:** 2.5 ± 0.6 |
| V  (L/kg ± SD) | **Vc:** 0.43 ± 0.18  **Vt:** 0.46 ± 0.46  **Vd_ss_:** 0.89 ± 0.64 | **Vc:** 0.28 ± 0.028  **Vt:** 0.24 ± 0.15**^b^**  **Vd_ss_:** 0.52 ± 0.44 | **Vc:** 0.39 ± 0.19  **Vt:** 0.037 ± 0.02  **Vd_ss_:** 0.43 ± 0.16 |
| K (h ± SD) | **K_12_:** 1.01 ± 0.22**^c^**  **K_21_:** 0.9 ± 0.5  **K_e_:** 1.2 ± 0.12 | **K_12_:** 1.4 ± 0.54**^b^**  **K_21_:** 1.6 ± 0.56  **K_e_:** 0.63 ± 0.15**^a^** | **K_12_:** 0.2 ± 0.3  **K_21_:** 2.2 ± 1.2  **K_e_:** 1.6 ± 0.56 |
| T_1/2_ (h ± SD) | **α** 0.25 ± 0.06  **β** 1.7 ± 0.9  **K_10_** 0.6 ± 0.05 | **α** 0.2 ± 0.08  **β** 2.3 ± 0.6**^b^**  **K_10_** 1.1 ± 0.3**^b^** | **α** 0.25 ± 0.4  **β** 0.5 ± 0.01  **K_10_** 0.4 ± 0.2 |
| AUC_0-24_  (μg*h/mL ± SD) | 2.8 ± 0.6  **N:** 0.99 ± 0.6 | 25.8 ± 2.1  **N:** 3.4 ± 0.2**^a, b^** | 2.4 ± 0.7  **N:** 0.97 ± 0.1 |
| AUC_0-∞_  (μg*h/mL ± SD) | 5.7 ± 1.8  **N:** 1.97 ± 0.33**^c^** | 43.7 ± 6.6  **N:** 5.7 ± 0.01**^a, b^** | 4.2 ± 0.9  **N:** 1.08 ± 0.4 |
| MRT (h ± SD) | 1.76 ± 0.2  **N:** 0.6 ± 0.02**^c^** | 3.0 ± 1.03  **N:** 0.4 ± 0.08 | 0.69 ± 0.6  **N:** 0.26 ± 0.2 |
| Cl _rate_  (mL/h/Kg ± SD) | **Cl_D_:** 437.1 ± 58.4**^c^**  **Cl_T_:** 505.3 ± 183.1 | **Cl_D_:** 394.4 ± 24.5**^b^**  **Cl_T_:** 174.1 ± 111.3**^a, b^** | **Cl_D_:** 81.3 ± 93.2  **Cl_T_:** 631.6 ± 17.4 |
| R^2^ | 0.8660 | 0.8710 | 0.8500 |

Statistical analysis with Brown-Forsythe and Welch ANOVA tests, with Dunnett’s T3 multiple comparisons (p<0.05). Significant differences between C16 with C15 are indicated with “a”. Significant differences between C16 with C17 are indicated with “b”. Significant differences between C15 with C17 are indicated with “c”. No significant differences detected for Vc, Vdss, K21, T1/2α, and MRT. N: Cp0, AUC, and MRT normalized for 1mg/mL dose.
