# Supplementary material for: TunR2, a novel mode-of-action tunicamycin-type antibiotic: Pharmacokinetics in C57BL/6 mouse and Holstein cattle
Source: PLoS One. 2025 Jul 23;20(7):e0327932. doi: 10.1371/journal.pone.0327932 (PMC12286339; doi:10.1371/journal.pone.0327932)
Supplement: S8 Table — (DOCX) [file pone.0327932.s025.docx]

**S8 Table**: **Map shedding in the feces by culture for 6-12 weeks on HEYM.**

| **Feces culture results** | **TunR2 group** | | | **Control**  **#12802** |
| --- | --- | --- | --- | --- |
|  | **5327** | **6739** | **6878** |  |
| Pre-treatment  (day 0) | N | L | VH | N |
| During the treatment  (day 3) | N | L | VH | N |
| Post-treatment  (end point day) | N | M | VH | N |

N=negative, L= low (1-10 CFU), M=moderate (11-50 CFU), and VH=very heavy (>100 CFU)
