# Supplementary material for: TunR2, a novel mode-of-action tunicamycin-type antibiotic: Pharmacokinetics in C57BL/6 mouse and Holstein cattle
Source: PLoS One. 2025 Jul 23;20(7):e0327932. doi: 10.1371/journal.pone.0327932 (PMC12286339; doi:10.1371/journal.pone.0327932)
Supplement: S9 Table — (DOCX) [file pone.0327932.s027.docx]

**S9 Table.** **Culture and PCR for the presence of Map in bovine tissues.**

| **Tissue qPCR and culture results** | **TunR2 group** | | | | | | **Control**  **#12802** | |
| --- | --- | --- | --- | --- | --- | --- | --- | --- |
|  | **5327** | | **6739** | | **6878** | |  |  |
|  | qPCR | Culture | qPCR | Culture | qPCR | Culture | qPCR | Culture |
| ICV + LN | S | N | VH | VH | VH | VH | L | N |
| Ileum + LN | S | L | VH | VH | VH | VH | M | N |
| Jejunum + LN | L | N | H | M | VH | VH | L | H |
| Spleen | VH | L | S | M | M | VH | S | L |
| Liver | L | L | L | VH | H | VH | L | N |
| Ovary | L | N | S | L | H | VH | L | L |
| Uterus | S | N | L | M | H | VH | S | L |
| Mammary gland | S | N | S | L | VH | VH | L | N |

ICV: ileocecal valve; LN: lymphonodes; N=negative (Ct > 40 or no CFU), S=suspect (Ct 40-36.1), L=(Ct 36-33.1 or 1-10 CFU), M=moderate (Ct 33-30.1 or 11-50 CFU), H= heavy (Ct 30-25.1 or 51-100 CFU), and VH=very heavy (Ct < 25 or CFU>100)
