# Supplementary material for: TunR2, a novel mode-of-action tunicamycin-type antibiotic: Pharmacokinetics in C57BL/6 mouse and Holstein cattle
Source: PLoS One. 2025 Jul 23;20(7):e0327932. doi: 10.1371/journal.pone.0327932 (PMC12286339; doi:10.1371/journal.pone.0327932)
Supplement: S1 Appendix — Pharmacokinetic parameters were calculated by two-compartmental using a linear regression analysis and residuals method to calculate using Microsoft Excel 2024 using standard equations. (DOCX) [file pone.0327932.s029.docx]

# **Supplementary material – S1 Appendix**

Bi-exponential equation:

$C=A .\exp\left( -\alpha t \right)+B .\exp\left( -\beta t \right)$ (S1)

where C is the concentration, t is the time after dosing, and A, B, α, and β are the

coefficients or exponents for two-compartment model.

Estimation of pharmacokinetics parameters:

$Plasmatic concentration at time zero (Cp_{0})=A+B$ (S2)

$K_{21}=\frac{A \beta+ B\alpha}{A+B}$ (S3)

$K_{12}= \frac{(AB){(\beta-\alpha)}^{2}}{\left( A+B \right)[\left( A\beta\right)+\left( B\alpha\right)]}$ (S4)

$K_{10}=K_{e}= \frac{\alpha\beta}{K_{21}}$ (S5)

where K_21_ and K_12_ are the rate constants of distribution, and K_10_=K_e_ is the elimination rate constant.

$t_{\frac{1}{2} \alpha}= \frac{0.693}{\alpha}$ (S6)

$t_{\frac{1}{2} \beta}= \frac{0.693}{\beta}$ (S7)

$t_{\frac{1}{2}}= \frac{0.693}{K_{10}}$ (S8)

where t_1/2_ is the half-life (α: fast or distribution phase; β: slow or elimination phase; K_10_: elimination).

$V_{c}=\frac{Dose (\frac{\mu g}{mL})}{{Cp}_{0}}$ (S9)

$V_{t}= \frac{V_{c} K_{12}}{K_{21}}$ (S10)

${Vd}_{ss}=V_{c} + V_{t}= \frac{K_{12}+K_{21}}{K_{21}} V_{c}= V_{c} (1+ \frac{K_{12}}{K_{21}} )$ (S11)

where V_c_ is the volume in central compartment, V_t_ is volume in tissue (or peripheral) compartment, and Vd_ss_ is volume of distribution at steady state.

${Cl}_{D}= K_{12}V_{c} =K_{21}V_{t}$ (S12)

${Cl}_{T}= K_{10}V_{c} = \frac{Dose}{{AUC}_{0-\infty}}$ (S13)

where Cl_D_ is the intercompartment clearance or distribution rate, Cl_T_ is the total clearance rate, and AUC_0-∞_ is the area under the curve from time zero to infinite

$MRT=\frac{{Vd}_{ss}}{{Cl}_{T}}= \frac{AUMC}{AUC}$ (S14)

where MRT refers to the mean residence time.
