# Supplementary material for: TunR2, a novel mode-of-action tunicamycin-type antibiotic: Pharmacokinetics in C57BL/6 mouse and Holstein cattle
Source: PLoS One. 2025 Jul 23;20(7):e0327932. doi: 10.1371/journal.pone.0327932 (PMC12286339; doi:10.1371/journal.pone.0327932)
Supplement: Attachment — Submitted filename: Response to Reviewers.docx [file pone.0327932.s032.docx]

Manuscript PONE-D-25-19128

Dear Reviewer,

Thank you for the time and effort that you dedicated to providing constructive feedback on our manuscript “TunR2, a novel mode-of-action tunicamycin-type antibiotic: pharmacokinetics in C57BL/6 mouse and Holstein cattle”. We incorporated the suggestions and hope that this version enhances our manuscript and could meet the PLOS one standards for publication. Please see below, in *blue*, the author answers to your comments point-by-point.

Reviewer #1 - Comments to the Author

Overall the manuscript is clearly presented, written well and presents data that justify the study claims and conclusions. Statistical analysis of the data appear to pass rigor and the data is available throughout the paper and supplemental materials. The mass spectrometry data does not appear to be fully available outside of the figures in the publication, but this may not be relevant to this study. Public repositories for storing MS raw files do exist for depositing large quantities of data.

There are a few grammatical errors and typos that need to be corrected during revision. Some examples include at line 143, 203, and 346. There are probably other errors that should be proofread for, as well. An additional oversight is the fact that the abstract states that non-compartmental PK analysis was used but it was established in the methods section of the paper and shown in supplemental figures that a two-compartment analysis was used to evaluate PK parameters. Another oversight appears to be Figure S3. Y-axis abundances are written as "Absorbance" but the figure legend indicates that the data is from ESI-MS which does not record absorbance.

*Author’s answer: we have reviewed and corrected typos and grammatical errors. We corrected the abstract and Figure S3.*

While the data presented are supported and discussed in the text, some of the discussion of the mass spectrometry standard samples seems unnecessary and does not contribute to the overall scientific body of knowledge, specifically, discussing the need to analyze standards for Tun, TunR1 and TunR2 dissolved in biological matrix. This is a well-known phenomenon in mass spectrometry and it is considered common practice to analyze standard curve points and QC samples that have been spiked into the biological matrix of interest. Unless this is a novel discovery specific to these Tunicamycin molecules that had not previously been published, this portion of the manuscript does not contribute to the overall impact of the paper. Additionally, the assertion that TunR2 passes into the feces structurally unmodified is an important claim. It is discussed in the text but no data is shown that backs up this assertion. Structural determination of various modifications is important and not easily done by mass spectrometry alone. If the authors wish to leave this assertion in the paper or discuss it, some data should be included that support this claim.

*Author’s answer: We agree with the reviewer that LC/MS is a fairly standard technique for the analysis of drugs, and we have therefore removed the specific details of the MS methodology. However, we found that selective ion monitoring (SIM) of [M + H - 221]+ fragment ion peaks was needed to give sufficient sensitivity. The Tun compounds were not detectable by standard electrospray MS monitoring and hence the MS-SIM technique requires some explanation. MALDI-TOF/MS is less commonly used to assay small molecules, but we found it useful for rapidly screening multiple TUN components simultaneously without prior chromatographic separation. We feel that some explanation for this is needed.*

*Also, we remove from the manuscript the statement that TunR2 passes unchanged since we only have LC-MS data as pointed by the reviewer.*

It would have been a nice addition to the paper to see a different beta-lactam drug besides oxacillin that could have been subjected to some sort of PK analysis. However, there could be many different reasons for the choice of oxacillin in the study and this is not a major concern.

*Author’s answer: We appreciate the reviewer’s insightful comment. Oxacillin was selected as the representative beta-lactam in combination with tunicamycin derivatives for in vivo PK evaluation because oxacillin had a very pronounced enhancement with TunR2 (32- to 64-fold) when we tested them together in vitro (Price et al, 2017). While we agree that the evaluation of the potential synergism with other beta-lactams and their PK would be of great interest for broadening the scope of synergistic interaction, our initial goal was to establish a proof-of-concept with a single, well-characterized beta-lactam antibiotic.*

*Citation: Neil PJ Price, Trina M Hartman, Jiakun Li, Kiran K Velpula, Todd A Naumann, Maheedhara R Guda, Biao Yu and Kenneth M Bischoff (2017). Modified tunicamycins with reduced eukaryotic toxicity that enhance the antibacterial activity of β-lactams. Journal of Antibiotics 70, 1070–1077.*

The manuscript thoroughly studies and establishes promise for Tunicamycin analogs to be used as adjuvants for antimicrobials. The data examine the PK/PD of Tun, TunR1, and TunR2 in a mouse model and look for toxic buildup of the analytes in off-target tissues and examine the administration of the experimental molecules with another antibiotic. The PK/PD analysis is thorough and well done. Next, Tun, TunR1, and TunR2 are given to cattle in a well-controlled study at sub-therapeutic doses to study tissue accumulation and potential side effects of these molecules. The rationale given for cattle dosing and the analysis of the various routes of elimination and potential tissue accumulation in the animals is well reasoned and well done. Overall, this study established the merits of these molecules as non-toxic alternatives to normally toxic Tunicamycin and successfully make the case that further study is needed of these promising analogs. Furthermore, the study clearly examines and establishes that of the Tun analogs, TunR2 is likely the more promising candidate, which is a result that is sure to inform future studies.

*Author’s answer: we sincerely thank the reviewer for their thorough and encouraging evaluation of our manuscript. We are grateful for the constructive feedback and support.*
